# Targeting *IRG1* in tumor-associated macrophages for cancer therapy

**DOI:** 10.1093/procel/pwaf012

**Published:** 2025-02-18

**Authors:** Shuang Liu, Lin-Xing Wei, Qian Yu, Zhi-Wei Guo, Chang-You Zhan, Lei-Lei Chen, Yan Li, Dan Ye

**Affiliations:** National Resource Center for Mutant Mice and MOE Key Laboratory of Model Animal for Disease Study, Jiangsu Key Laboratory of Molecular Medicine, Chemistry and Biomedicine Innovation Center (ChemBIC), Model Animal Research Center, Department of Oncology, Nanjing Drum Tower Hospital, Affiliated Hospital of Medical School, Nanjing University, Nanjing 210061, China; Shanghai Key Laboratory of Clinical Geriatric Medicine, Shanghai, Huadong Hospital, and Shanghai Key Laboratory of Medical Epigenetics, International Co-laboratory of Medical Epigenetics and Metabolism (Ministry of Science and Technology), and Molecular and Cell Biology Lab, Institutes of Biomedical Sciences, Shanghai Medical College of Fudan University, Shanghai 200040, China; National Resource Center for Mutant Mice and MOE Key Laboratory of Model Animal for Disease Study, Jiangsu Key Laboratory of Molecular Medicine, Chemistry and Biomedicine Innovation Center (ChemBIC), Model Animal Research Center, Department of Oncology, Nanjing Drum Tower Hospital, Affiliated Hospital of Medical School, Nanjing University, Nanjing 210061, China; Department of Pharmacology, School of Basic Medical Sciences & Department of Pharmacy, Fudan University, Shanghai 200032, China; Department of Pharmacology, School of Basic Medical Sciences & Department of Pharmacy, Fudan University, Shanghai 200032, China; Shanghai Key Laboratory of Clinical Geriatric Medicine, Shanghai, Huadong Hospital, and Shanghai Key Laboratory of Medical Epigenetics, International Co-laboratory of Medical Epigenetics and Metabolism (Ministry of Science and Technology), and Molecular and Cell Biology Lab, Institutes of Biomedical Sciences, Shanghai Medical College of Fudan University, Shanghai 200040, China; National Resource Center for Mutant Mice and MOE Key Laboratory of Model Animal for Disease Study, Jiangsu Key Laboratory of Molecular Medicine, Chemistry and Biomedicine Innovation Center (ChemBIC), Model Animal Research Center, Department of Oncology, Nanjing Drum Tower Hospital, Affiliated Hospital of Medical School, Nanjing University, Nanjing 210061, China; Shanghai Key Laboratory of Clinical Geriatric Medicine, Shanghai, Huadong Hospital, and Shanghai Key Laboratory of Medical Epigenetics, International Co-laboratory of Medical Epigenetics and Metabolism (Ministry of Science and Technology), and Molecular and Cell Biology Lab, Institutes of Biomedical Sciences, Shanghai Medical College of Fudan University, Shanghai 200040, China; Department of General Surgery, Huashan Hospital, Fudan University, Shanghai 200040, China

**Dear Editor,**

A hallmark of immune cells in response to inflammatory stimuli, both infectious and non-infectious, is the rapid and extensive change in metabolism. In addition to meet the energetic and biosynthetic need of the immune cell, research over the past decade has led to the appreciation of individual metabolites in cell signaling during metabolic reprogramming in immune response. One illustrative example is itaconate, a dicarboxylic acid derived from Kreb cycle metabolite, cis-aconitate, by the enzyme cis-aconitate decarboxylase (ACOD1). ACOD1 is encoded by immunoresponsive gene 1 (IRG1), which is rapidly induced by various inflammatory stimuli in myeloid cells, leading to the prompt accumulation of itaconate to millimolar levels. Itaconate binds to multiple proteins to influence oxidative response, epigenetic modification, and gene expression intrinsically and to signal GPCR after secretion (Ye et al., 2024). These regulations on different pathways by a single metabolite concertedly modulate inflammatory responses. Beyond its role as an antimicrobial metabolite, itaconate has recently garnered attention in the research of cancer biology. Genetic and preclinical studies in animal models suggest that itaconate can create an immunosuppressive tumor microenvironment. The enhanced antitumor immunity and response to immune checkpoint inhibitors seen in *Irg1*-deficient mice, which otherwise exhibit normal development, underscore IRG1 as a compelling target for cancer immunotherapy (Chen et al., 2023; Gu et al., 2023; Zhao et al., 2022). However, current strategies to target IRG1/itaconate remain limited, emphasizing the need for therapeutic development to effectively blockade IRG1 in cancer treatment.

RNA therapies, including small interfering RNA (siRNA), offer a potent strategy to modulate gene expression by specifically degrading target mRNA, thus preventing the production of disease-associated proteins. In this study, we utilized siRNAs to selectively deplete mouse and human *IRG1* gene in mouse bone marrow derived macrophages (mBMDMs) and THP1-derived human macrophages, respectively (Fig. S1). We previously demonstrated that itaconate, produced by IRG1, inhibits the activity of TET2 DNA dioxygenase to suppress the expression of inflammatory genes, such as *CXCL9* and *CXCL10*. These chemokines bind to their receptor CXCR3 on T cells and promote CD8^+^ T cell chemotaxis (Chen et al., 2023). In line with earlier findings in *Irg1*-deficient macrophages (Chen et al., 2022b; Mills et al., 2018), si*Irg1* electro-transfection led to upregulation of inflammatory cytokine and chemokine genes, such as *Il1b*, *Il6*, and *Cxcl9*, in mBMDMs following exposure to tumor conditioned medium (TCM) from B16-F10 melanoma cells (Fig. S2A). Similarly, transfection of si*IRG1* upregulated the mRNA expression of *CXCL9*, *CXCL10*, and *CXCL11* in human macrophages exposed to TCM from MDA-MB-231 breast cancer cells (Fig. S2B). This knockdown enhanced the ability of human macrophages to promote Jurkat T cell migration in a co-culture transwell system (Fig. S2C). These findings suggest that targeting *IRG1* via siRNAs can modulate macrophage function, potentially enhancing antitumor immune responses.

Lipid nanoparticles (LNPs), mainly composed of ionizable lipids, cholesterol and polyethylene glycol (PEG)-lipids, have emerged as a prominent delivery system for nucleic acid therapeutics (Cullis and Hope, 2017). Building upon the success of Patisiran, a clinically approved siRNA therapy utilizing LNP technology (Akinc et al., 2019), we developed an LNP-mediated delivery system for siRNAs targeting *IRG1* gene. The formulated LNPs exhibited hydrodynamic diameters of approximately 56.6 nm for siNC and 58.6 nm for si*Irg1*, with narrow polydispersity indices of 0.030 ± 0.013 and 0.045 ± 0.024, respectively, indicating uniform particle sizes (Fig. S3A and S3B). The zeta potentials were slightly negative, and the encapsulation efficiencies were high, at 96.957% for si*NC* and 96.870% for si*Irg1*, ensuring effective nucleic acid protection and a substantial process yield (Fig. S3C and S3D). In mBMDMs stimulated with TCM from B16-F10 or E0771 cells, treatment with LNP-si*Irg1* achieved knockdown efficiencies of 61.1% and 57.5%, respectively (Fig. S3E). This knockdown led to the upregulation of chemotaxis genes, such as *Cxcl9* and *Cxcl10*, in mBMDM challenged with B16-F10-TCM (Fig. S3F). Consequently, LNP-si*Irg1* enhanced the ability of mouse macrophages to facilitate CD8^+^ T cell migration in the aforementioned co-culture transwell system (Fig. S3G).

*In vivo* studies using a syngeneic mouse tumor model, established by subcutaneously inoculating MC38 colon cancer cells, demonstrated that a single intravenous injection of labeled LNPs targeted approximately 15% of tumor-associated macrophages (TAMs) in tumor-bearing immunocompetent mice (Fig. S3H and S3I). Moreover, three intravenous injections of LNP-si*Irg1* led to a significant depletion of *Irg1* mRNA in TAMs by about 56.0% in MC38 tumor-bearing mice (Fig. 1A and 1B). This reduction corresponded to decreased intracellular levels of itaconate in TAMs by 38.6% and 76.4% in mice bearing MC38 and B16-F10 tumors, respectively (Fig. 1C). Notably, LNP-si*Irg1* efficiently inhibited tumor growth and reduced tumor weight by approximately 57.7% in MC38 and 75.4% in B16-F10 tumor models (Fig. 1D–I). These results thus underscore the potential of LNP-si*Irg1* as a promising cancer therapeutic in immunocompetent mice.

**Figure 1.**
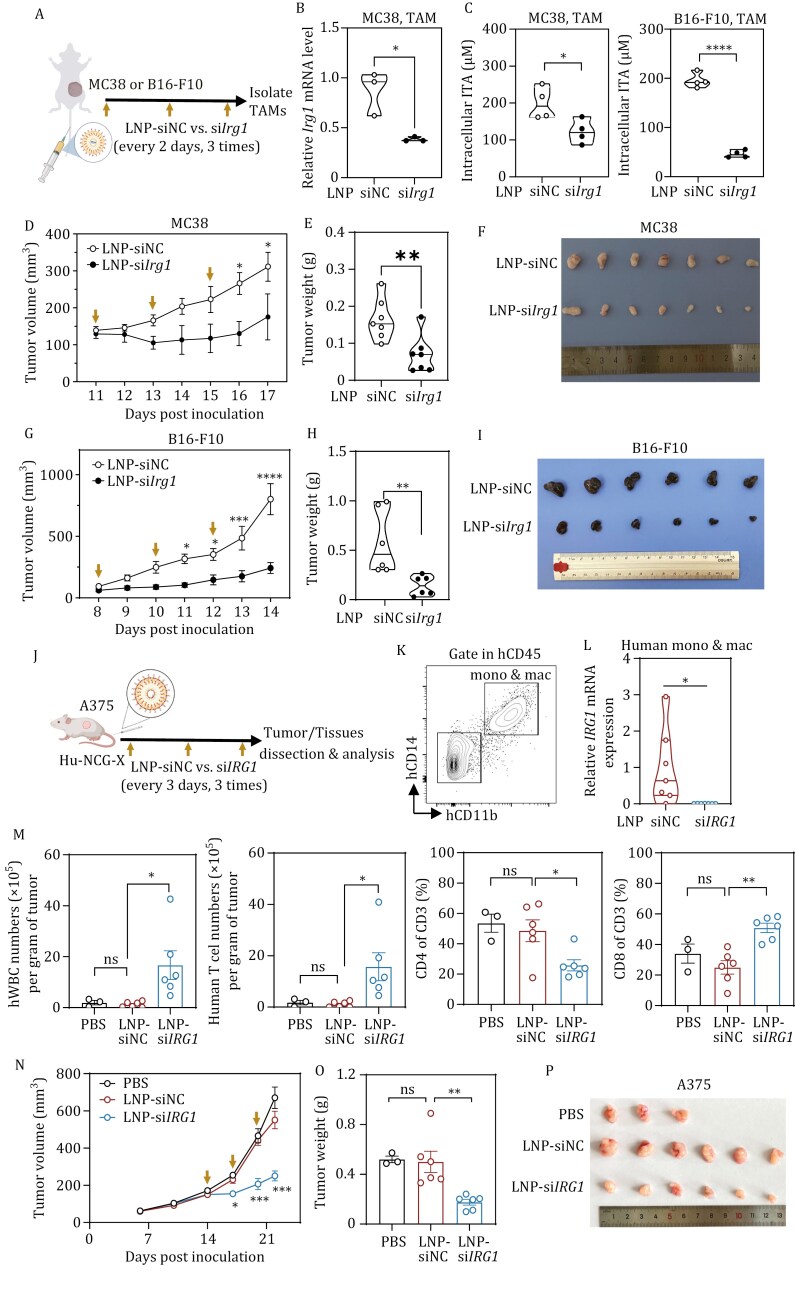
**Targeting *IRG1* inhibits tumor growth in HIS mice**. (A) Experimental schematics of LNP-si*Irg1* therapy in immune competent mice. Briefly, MC38 or B16-F10 was inoculated subcutaneously. When the tumor volume reached about 100 mm^3^, LNP-siNC and LNP-si*Irg1* were injected intravenously for 3 times, every 3 days. Tumors were then dissected and analyzed. (B and C) TAMs were isolated from tumor described in (A) by flow cytometry. The knockdown efficiency of *Irg1* in TAMs was determined by qRT-PCR (*n* = 3 per group; B). The intracellular itaconate in TAMs was determined by LC-MS analysis (*n* = 4 per group; C). (D and G) Tumor growth of immunocompetent mice described in (A) was measured and recorded (*n* = 6–7 per group). (E and H) Tumor weight of immunocompetent mice described in (A) (*n* = 6–7 per group). (F and I) Representative tumor images were shown. (J) Experimental schematics of LNP-si*IRG1* therapy in HIS mice bearing A375 melanoma (*n* = 3–6 per group). Briefly, A375 was inoculated subcutaneously. After 2 weeks, PBS, LNP-siNC, and LNP-si*IRG1* were injected intravenously for 3 times, every 3 days. Tumors and tissues were then dissected and analyzed. (K and L) Tumor-infiltrating human mono & mac (hCD45^+^hCD14^+^hCD11b^+^) were isolated by flow cytometry (K). The mRNA expression of *IRG1* was determined by qRT-PCR (L) (*n* = 7 per group). (M) Tumor-infiltrating immune cells described in (J) were analyzed by flow cytometry. Numbers of hCD45^+^ and T cells and the proportion of CD4^+^ or CD8^+^ T cells in CD3^+^ cells in melanoma samples were detected by flow cytometry (*n* = 3–6 per group). (N) Tumor growth of HIS mice described in (J) was recorded. (O and P) A375 melanoma samples were dissected and weighted after LNP-siRNA therapy (O) (*n* = 3–6 per group). Representative tumor images were shown (P). Data are mean ± SEM. The *P* values were calculated by unpaired, two-tailed Student’s *t*-test for (B, C, D, E, G, H, and L) and were calculated by one-way ANOVA for (M, N, and O). *P < 0.05, ** *P* < 0.01, *** *P* < 0.001, **** *P* < 0.0001 and ns, non-significant (*P* ≥ 0.05).

Species differences in hematopoiesis and immunity necessitate the use of human immune system (HIS) mouse models for the development and evaluation of novel therapeutics (Allen et al., 2019; Chuprin et al., 2023). A well-established HIS mouse model is generated by the transplantation of human hematopoietic stem cells (HSCs) into severely immunocompromised newborn pups. This process allows the functional reconstitution of multiple human hematopoietic lineages, making this model indispensable for human cancer research (Chen et al., 2022a; Luo et al., 2023; Zhai et al., 2023). To assess the antitumor potential of LNP-si*IRG1* in human, we developed HIS mice using NCG-X (NOD-Prkdc^em26Cd52^Il2rg^em26Cd22^ kit^em1Cin(V831M)^/Gpt) recipients (Fig. S4A). The NCG-X strain is genetically modified from the NCG immunodeficient mice with a mutant *Kit* allele to permit human HSC engraftment in nonconditioned recipients and enhanced myeloid cell and T cell differentiation, offering advantages over standard irradiated NCG recipients (Cosgun et al., 2014). We monitored the reconstitution of human immune cells by analyzing blood samples from HIS mice at different time points post human HSC engraftment. After 12 weeks, human white blood cells (hWBCs) in the peripheral blood reached approximately 2.5 × 10^5^/mL, accounting for ~32.0% of total WBCs (Fig. S4B and S4C). T and B cells accounted for ~13.7% and ~75.5%, respectively, of human CD45^+^ immune population (Fig. S4D), while human CD14^+^ monocytes/macrophages (mono & mac) and CD56^+^ NK cells made up around ~1.6% and ~0.5%, respectively (Fig. S4E). The balance between CD4^+^ and CD8^+^ T cells was maintained, with naïve T cells representing ~58.8% of total human CD3^+^ T cells (Fig. S4F and S4G). To further explore immune cell dynamics in the tumor microenvironment (TME), we then evaluated the hCD45^+^ tumor infiltrated lymphocytes (hTILs) in HIS mice bearing A375 human melanoma tumors. We found that human mono & mac and T cells comprised approximately 13.6% and 61.7% of total hTILs, respectively, distinct from those ratios in the peripheral blood (Fig. S4H–J). Collectively, these results confirm the successful reconstitution of human immune cells in NCG-X mice and highlight their potential for investigating human TME interactions and immune responses.

Next, the antitumor efficacy of LNP-si*IRG1* was determined in HIS mice bearing A375 human melanoma (Fig. 1J). After three intravenous injections, tumors were dissected and infiltrating human monocytes and macrophages were isolated. Strikingly, LNP-si*IRG1* dramatically reduced *IRG1* mRNA expression by 99.7% in human monocytes and macrophages in the TME (Fig. 1K and 1L). Further analysis of the TME revealed that LNP-si*IRG1* therapy significantly increased the infiltration of hWBCs and total T cells, particularly CD8^+^ cytotoxic T cells (Fig. 1M), without affecting the ratio of hWBCs and T cells in the spleen of HIS mice (Fig. S6A). Despite this increase in CD8^+^ T cell infiltration, LNP-si*IRG1* did not alter T cell function in either the melanoma or spleen as compared to LNP-siNC and PBS groups (Figs. S5 and S6B–E). Notably, LNP-si*IRG1* significantly suppressed human melanoma growth in HIS mice (Fig. 1N), reducing tumor weight by ~64.9% compared with the LNP-si*NC* group (Fig. 1O and 1P). These findings are consistent with our previous findings in *Irg1*-deficient immunocompetent mice (Chen et al., 2023), and reinforce the notion that targeting *IRG1* present a promising therapeutic strategy for cancer treatment, not only in mouse models but also in the context of the human immune system.

Finally, we evaluated the safety of LNP-si*IRG1 in vivo*. The administration of LNP-si*IRG1* didn’t affect the body weight of HIS mice (Fig. 2A). Systemic inflammation, as indicated by the levels of cytokines and chemokines in peripheral blood, remained largely unchanged following LNP-si*IRG1* treatment (Fig. 2B). Histopathological analysis revealed no obvious tissue injury in the lung, heart, kidney, liver, and spleen after LNP-si*IRG1* treatment compared to LNP-si*NC* and PBS groups (Fig. 2C–H). In addition, we also assessed liver function, liver damage, and kidney function of HIS mice through blood biochemistry analysis. Our data illustrated that LNP-si*IRG1*-treated HIS mice displayed comparable results to those of LNP-si*NC* and PBS controls, indicating no systemic toxicities (Fig. S7). Furthermore, LNP-si*IRG1* treatment did not induce additional immune cell infiltration in the tissues of HIS mice, including the lung, heart, kidney, liver, and spleen, compared with LNP-si*NC* and PBS controls (Fig. S8). Taken together, our results indicate that LNP-si*IRG1* enhances antitumor immunity by increasing the infiltration of lymphocytes and cytotoxicity T cells, without triggering systemic inflammation or causing tissue damage.

**Figure 2.**
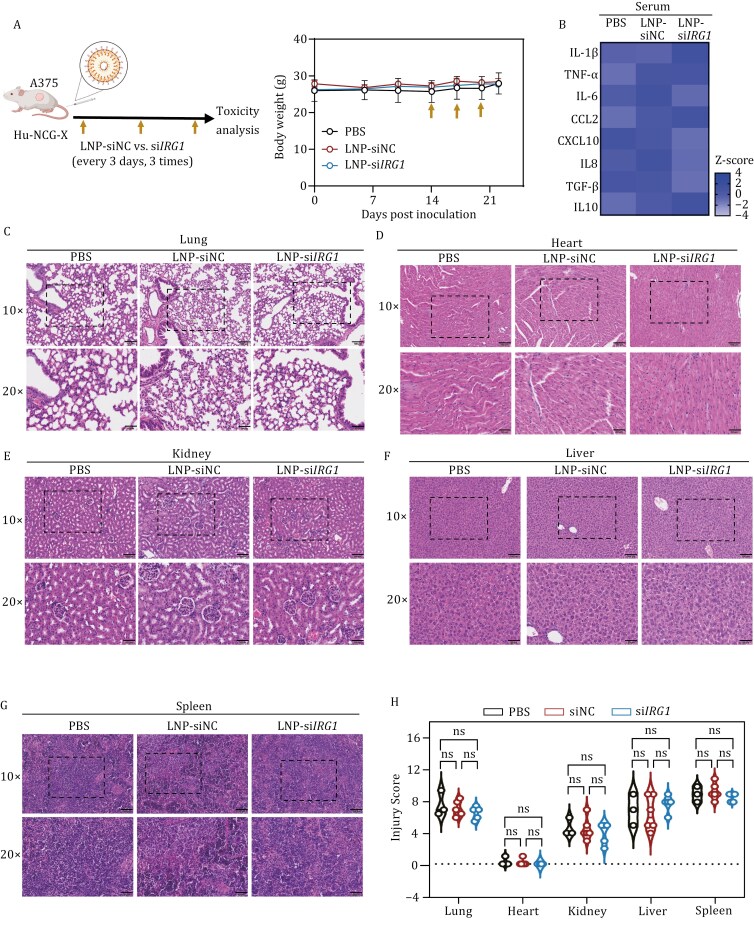
**Lipid nanoparticle-based delivery of si*IRG1* is biocompatible and safe**. (A) Experimental design of safety assessment of LNP-si*IRG1* therapy in HIS mice bearing A375 melanoma. The body weight of HIS mice was recorded throughout the experiment. (B) The secreted protein levels of indicated cytokines and chemokines were measured/standardized and exhibited by heatmap. Human cytokines in the plasma of HIS mice were determined by beads-based Legendplex assay. (C–G) Images are representative hematoxylin & eosin (H&E)-stained sections of indicated organs from HIS mice described in (A). Scale bars represent 100 μm (original magnification 10×) and 50 μm (original magnification 20×). (H) Histological scoring of H&E-stained tissues was quantified. Data are mean ± SEM, and the *P* values were calculated by one-way ANOVA. ns denotes non-significant (*P* ≥ 0.05).

As a prime example of metabolic rewiring in innate immunity, the expression of IRG1 is induced specifically in cells of myeloid lineages, leading to high accumulation of itaconate. The tumor-promoting activity of this metabolite was reported by recent studies: Zhao et al. (Zhao et al., 2022) reported that intracellular itaconate was higher in polymorphonuclear myeloid derived suppressor cells (PMN-MDSCs) than naïve bone marrow cells, and they proposed a non-cell-autonomous mechanism where itaconate produced by myeloid cells is secreted out, up-taken by T cells, and then attenuates CD8^+^ T cell proliferation and function. In another study, Gu et al. (Gu et al., 2023) reported itaconate promoted hepatocellular carcinoma progression by promoting succinate-dependent epigenetic induction of CD8^+^ T cell exhaustion. Unlike the non-cell-autonomous mechanism, we (Chen et al., 2023) previously reported a cell-autonomous mechanism in which itaconate produced by myeloid cells regulates gene expression, thereby altering their inflammatory features and potential roles in recruiting CD8^+^ T cells into tumor sites. Moreover, we demonstrated the tumor-promoting effects of *Irg1*/itaconate are primarily mediated by macrophages, rather than neutrophils. In this study, we employed a clinically approved LNP-siRNA therapy to deplete *IRG1* in TAMs and confirmed the increased infiltration of cytotoxic CD8^+^ T cells without altering their functionality or exhaustion. Despite different cell types and mechanisms of itaconate action, this metabolite creates an immunosuppressive TME to support tumor growth. Supporting the notion that targeting IRG1 has anti-neoplastic properties, Gu et al. show that ibuprofen, a broad-spectrum nonsteroidal anti-inflammatory drug, can prevent the induction of *Irg1* expression in activated macrophages through the inhibition of NF-κB signaling pathway. In this study, we provided both *in vitro* and *in vivo* evidence that specifically targeting *IRG1* gene by an LNP-mediated siRNA delivery strategy can enhance antitumor immunity in both immunocompetent mice and HIS mice with relatively high safety and efficacy.

In summary, our works pave the way for further development of IRG1-targeting therapies for cancer treatment. While our results in the melanoma model are promising, further research is needed to assess the effectiveness of LNP-si*IRG1* in other cancer types and across different tumor microenvironments. Clinical translation and validation of LNP-si*IRG1* are also warranted to evaluate its therapeutic potential in human patients, including dose optimization, delivery efficiency, and long-term safety.

## Supplementary data

Supplementary data is available at *Protein & Cell* online https://doi.org/10.1093/procel/pwaf012.

pwaf012_suppl_Supplementary_Materials

## Footnotes

We thank Hao-Yu Chen, Shi-Chao Zhang, Jia-Le Qian, and Li-Ping Meng (Crystal Pharmatech) for providing technique supports with LNP formulation and package. We also thank the staff members working at the Core Facility of Shanghai Medical College, Fudan University for their help with the mass spectrometry analyses. This study is supported by grants from Noncommunicable Chronic Diseases-National Science and Technology Major Program (No. 2023ZD0500400 to D. Y. and Y. L.), the National Key R&D Program of China (No. 2020YFA 0803202 to D. Y. and No. 2019YFA 0802900 to Y. L.), the Fundamental Research Funds for the Central Universities (No. 2024300408 to Y. L.), the Jiangsu Provincial Science and Technology Plan Special Fund (No. BK20232018 to Y. L.) and NSFC grants (Nos. 82225036, 31821002, and 31871431 to

D. Y.; Nos. 82103366 and 32370823 to L-L. C.; Nos. 32122035 and 32471000 to Y. L.). Y. L. is currently consulting for GemPharmatech Co.

L.L.C., Y.L., and D.Y.; methodology: S.L., L.X.W., Z.W.G., Q.Y., L.L.C., Y.L., and D.Y.; formal analysis: L.L.C. and D.Y.; investigation: S.L., L.X.W., Z.W.G., Q.Y., L.L.C., Y.L., and D.Y.; resources: Y.L. and D.Y.; writing of the original draft: L.L.C. and D.Y.; review and editing of the manuscript: L.L.C., Y.L., and D.Y.; supervision: L.L.C., Y.L., and D.Y.; and funding acquisition: L.L.C., Y.L., and D.Y. All authors declare that they have contributed to the study and approve the content of this manuscript. All authors give their consent for the publication of this manuscript in *Protein & Cell*. The authors affirm that the manuscript is original, has not been published elsewhere, and is not under consideration by another journal.

The authors declare no conflict of interest.

All mice were maintained under specific pathogen-free conditions and handled according to the Animal Ethics Committee of Shanghai Medical College, Fudan University (AP#: 2022JSIBS-012) and the Institutional Animal Care and Use Committee at Model Animal Research Center of Nanjing University (AP#: LY-01, LY-03).

## Notes

All unique/stable reagents in this study are available from the lead contact with a completed Materials Transfer Agreement. The processed data and the raw data are available upon request. Any additional information required to reanalyze the data reported in this paper is available from the lead contact upon request.
