# Supplementary material for: Targeting IRG1 in tumor-associated macrophages for cancer therapy
Source: Protein Cell. 2025 Feb 18;16(6):478–83. doi: 10.1093/procel/pwaf012 (PMC12187510; doi:10.1093/procel/pwaf012)
Supplement: pwaf012_suppl_Supplementary_Materials [file pwaf012_suppl_supplementary_materials.pdf]

MATERIALS AND METHODS

Animals

Female C57BL/6 mice were purchased from Beijing Vital River Laboratory Animal Technology Co., Ltd. (Shanghai, China). Animals were given unrestricted access to a standard diet and water. Animal experiments were performed at Fudan Animal Center in accordance with animal welfare guidelines, and the procedures were approved by the Ethics Committee of the Institutes of Biomedical Sciences (IBS), Fudan University (2022JSIBS-012). Immune deficient NOD Prkdc^{em26Cd52}Il2rg^{em26Cd22}Kit$^{em1Cin(V831M)}$/Gpt (NCG-X) mice were purchased from GemPharmatech Co (Nanjing, China). NCG-X mice were housed in a specific pathogen-free (SPF) microisolator environment with humane care. The Institutional Animal Care and Use Committee (IACUC) at Model Animal Research Center of Nanjing University approved all HIS animal procedures used in this study (AP#: LY-01), which are complied with all relevant ethical regulations.

Cell culture

MC38 cells (Kerafast, catalog no. ENH204), B16-F10 cells (ATCC, CRL-6475), E0771 cells (ATCC, CRL-3461), MDA-MB-231 (ATCC, HTB-26), and A375 (ATCC, CRL-1619) were commercially purchased and cultured in Dulbecco's modified Eagle's medium (DMEM, Hyclone, Cat# SH30243.02) containing 10% fetal bovine serum (FBS) (ExCell Bio, Cat# FSD500) and 1% penicillin/streptomycin antibiotics. THP1 (ATCC, TIB-202) and Jurkat T cells (ATCC, TIB-152) were maintained in RPMI1640 (Biological Industries, Cat# 01-100-1ACS) containing 10% FBS (GBICO, Cat#: 10091148) and 1% penicillin/streptomycin antibiotics.

For the differentiation of BMDMs, mice were euthanized in a 5% CO_2 chamber and death was confirmed by cervical dislocation. Bone marrow was harvested from the femur and the tibia and differentiated in DMEM containing 10% FBS, 1% penicillin/streptomycin, and mouse macrophage colony-stimulating factor (M-CSF, Sinobiology, Cat# 151112-M08H) (100 ng/ml) for 7 days.

Preparation of tumor cell conditioned medium

To prepare the tumor conditional medium, B16-F10, E0771, or MDA-MB-231 tumor cells were seeded with 10 ml medium in 100 mm dish. The culture medium was collected after 24 hours by filtration using 0.45-μm filter (Corning, Cat# 431225).

Generation of mouse tumor models

C57BL/6 mice were injected subcutaneously in the right flank with 2×10^5 MC38 or B16-F10 tumor cells in 25 μL PBS and 25 μL Matrigel (BD Biosciences, Cat# 356234). Tumors were measured every 2 or 3 days, and the volume was calculated using the formula V = as (width by width by length)/2 as previously described (Li et al., 2022).

Preparation of siRNA-loaded LNPs

LNPs were prepared by Crystal Pharmatech, as previously described (Akinc et al., 2019). Briefly, lipid components and RNA were first dissolved in organic phase and aqueous phase

to achieve a 1:3 volume ratio. Commercial ionized lipids DLin-MC3/CHOL/DSPC/PEG2000-DMG-OH at a ratio of 50/10/38.5/1.5 were dissolved in ethanol. siRNA was diluted in 25 mM sodium acetate. The final ionizable lipids: mRNA weight ratio was 10:1. Prepared solutions were mixed via a microfluidic mixing device at a flow rate of 12 mL/min to obtain LNPs. Then, prepared LNPs were dialyzed against PBS using 3500 MWCO (Molecular weight cut-off) dialysis cassettes (Life Technologies, Cat# 66110) overnight. LNPs were then characterized for their size and RNA therapeutics encapsulation efficiency.

Preparation of DiI-labeled LNPs

During the synthesis of LNP, DiI was added to the lipid solution (organic phase) at a concentration of 0.5 mol% (total lipid) to label the LNP.

Quantitative real-time PCR

Total RNA was extracted from cells using RNA purification kit (EZBioscience, Cat# B0004DP) and converted into complementary DNA (cDNA) using reverse transcription kit (EZBioscience, Cat# EZB-RT2GQ) as recommended by the manufacturer's instruction. Diluted cDNA was then used in RT-PCR reactions containing SYBR Premix and gene-specific primers. The reactions were performed in the QuantStudioTM 6 Flex Real-Time PCR System (Applied Biosystems). Actin was used as a housekeeping control. Primers used in this study are listed in **Table S1**.

Flow cytometry

Tumors were harvested at indicated time. Briefly, 100 mm^3 of each tumor was chopped, digested, and filtered through a 70-μm cell strainer to generate a single-cell suspension. TILs were enriched using Percoll (Cytiva, Cat# 17089109) density gradient centrifugation. After red blood cell lysis (Beyotime, Cat# C3702), cells were counted and plated in phosphate-buffered saline (PBS) containg 2% FBS and 2mM EDTA. Cells were blocked with human IgG (Sigma-Aldrich, Cat# I8640; 5 μg/ml, for HIS mice samples) or anti-mouse CD16/32 (Biolegend, Cat# 156604, for murine samples) followed by the staining of Fixable viability dye Eflour 506 (eBioscience, Cat# 65-0866-18; 1:800). Surface molecule staining was performed at 4°C for 15 to 30 min in PBS in the dark. For intracellular cytokines or human FOXP3 detection, freshly isolated cells were stimulated with 50ng/ml PMA (BioGems, Cat# 1652981) and 1μg/ml ionomycin (BioGemc, Cat# 5608212) for 4h in the presence of 5μg/ml brefeldin A (BioGems, Cat# 2031560) before intracellular cytokine and FOXP3 Staining. Buffer for the staining of FOXP3 was commercially purchased (eEbioscience, Cat# 00-5523-00). An NovoCyte Penteon flow cytometer (Aglient) was used to acquire data and FlowJo V10.62 software (TreeStar) was used for data analysis. Gating strategy for flow cytometry analysis is shown in **Fig. S9**. Antibodies used for flow cytometry is shown in **Table S2**.

T cell migration assay

T cell migration assay was performed by using 24-well 6.5-mm transwell with 5.0 μm Pore Polycarbonate Membrane Insert as previously reported (Chen et al., 2023). In brief, BMDMs (5×10^5) were treated with LNP-siRNA for 24 hours followed by the challenge of B16-F10-TCM for another 12 hours at the bottom chamber. $CD8^+$ T cells (1×10^6) were isolated from

the spleen of wild-type mice and suspended in 200 μl of 10% FBS–RPMI 1640 and were then placed at the top chamber. The migration of $CD8^+$ T cells from the top to the bottom chamber was evaluated after incubation for 4 hours at 37°C in a 5% CO_2 atmosphere. Cells in the bottom chambers were collected and the number of $CD8^+$ T cells were counted. Likewise, THP1 cells (5×10^5) were seeded at the bottom chamber and differentiated to macrophages by the treatment of PMA for 48 hours. siRNA was transfected in THP1-derived macrophages by RNAimax (Invitrogen, Cat# 13778150) followed by the challenge of MDA-MB-231-TCM for 12 hours. Jurkat cells (1×10^6) were suspended in 200 μl of 10% FBS–RPMI 1640 and were then placed at the top chamber. The migration of Jurkat cells from the top to the bottom chamber was evaluated after incubation for 4 hours at 37°C in a 5% CO_2 atmosphere.

Purification of hematopoietic stem cells (HSCs)

Human $CD34^+$ cells described in this article were obtained from fetal liver (FL) under clinical ethical approval from the ethical committee of Drum Tower Hospital with informed consent (protocol#: 2021-488-01). Two different donors of human FL $CD34^+$ cells were used in this study. Briefly, samples were mechanically dissociated and filtered through 70 μm sterile cell strainers (Jet Bio-Fil, Cat# CSS01370) followed by the Ficoll isolation (GE Healthcare, Cat# 17544203). HSCs were then enriched with the CD34 Microbead Kit (Miltenyi Biotec, Cat# 130-046-702). Purified cells were stained with antibodies for checking purity and were cryopreserved within 2 hours after collection in FBS/DMSO (7.5%) (Sigma-Aldrich, Cat# D2650).

Generation of human immune system (HIS) mice.

Generation of HIS mice were described as previously reported (Li et al., 2018; Ren et al., 2022). In brief, 3 to 7- day-old newborn NCG-X pups were injected intrahepatically with 5×10^4 human $CD34^+$ cells in 30 μL RPMI 1640 with a 29-gauge needle (BD bioscience, Cat# 328421). HIS mice with more than 1×10^5 human $CD45^+$ cells/mL of peripheral blood at week 10 post engraftment were used in this study.

Generation of tumor-bearing NCG-X HIS mice

12-week-old NCG-X HIS mice were inoculated subcutaneously in right flank with 5×10^6 A375 cells in 0.1 ml of RPMI 1640. Mice were randomly divided into three groups when the tumor volume reached about 200 mm^3. Mice received intravenous injection of PBS, LNP-siNC or LNP-si*IRG1* every three days from D14 to D21. After 3 times of injection, tumor cells were harvested for flow cytometry analysis following the aforementioned method.

Multiplex quantification of human cytokines

Blood was collected from the submandibular vein of HIS mice and plasma was collected through centrifugation of blood. Levels of various human cytokines in the plasma were measured using beads-based Legendplex Cytometry Array (BioLegend, Cat# 740930) according to manufacturer’s instruction. Data were acquired on NovoCyte Penteon flow cytometer and were analyzed using Legendplex software.

Biochemical detection

Tumor-bearing HIS mice were euthanized and plasma was collected through centrifugation of peripheral blood. Level of various biochemical indicators were analyzed by automated BX-3010 chemistry analyzer (Sysmex) according to the manufacturer's instruction.

Histopathology and immunohistochemistry

For immunohistochemistry, tissues were fixed in Animal Testicular Tissue Fixative Solution (Servicebio, Cat# G1121) and embedded in paraffin. Paraffin blocks were sectioned at 5 μm and stained with anti-human CD45 antibody (Abcam, Cat# AB8216; 1:200). Anti-mouse HRP conjugated secondary antibody (Jackson Immuno Research, Cat# 115-035-003; 1:500) was used according to manufacturer's instruction. Images were acquired using VS200 (Olympus) and analyzed by IHC Profiler of Image J software.

For Hematoxylin & Eosin staining, sections were stained with H&E, and evaluated by a pathologist who was blinded of the samples' identity. Scoring criteria of different organs were described as previous reports (Kleiner et al., 2005; Miao et al., 2010; Gauger et al., 2012; Gibson-Corley et al., 2013; Standage et al., 2016; Bajema et al., 2018).

Supplemental Fig. legend

A

	Position		siRNA sequence (sense)	Knockdown efficiency (Protein level)	Knockdown efficiency (mRNA level)	Targeting *IRG1* gene
1	1502	CDS	CAGGUUUACCAAUAUCUAAUU	99.1	90.5	Mouse
1	444	CDS	GCUGGCUUUCAAUGUUGGUAUUGAA	80.2	90.6	Human
2	1130	CDS	CACAGGUGAGAGAGCUGCUCAGUAA	50.4	85.4	Human
3	1345	CDS	UGGGACACAGUGGAAAGCCUUAUAA	59	68.8	Human
4	231	CDS	CAGUUCCAACAUAUCCAGCACUGUU	62.3	77.7	Human
5	986	CDS	GGAUACCAAAUGUCCAGUAUGUAAA	79.2	80.5	Human
6	432	CDS	CCUUGACCUGCUGCUGGCUUUCAAU	84.8	67.1	Human
7	722	CDS	UGGCAAUGUUGGGUCUCCAAGGAAA	81.1	80.3	Human
8	946	CDS	GCCCUGCUUCCAACUGACUACAUUA	86.9	73.2	Human
9	1630	3'UTR	UAUCUGGAAGGAGCCUUCUCCUGAA	68.7	79.5	Human
10	1731	3'UTR	GGGUGAAAUUCAACUCACCUGUGAU	47.9	83.1	Human

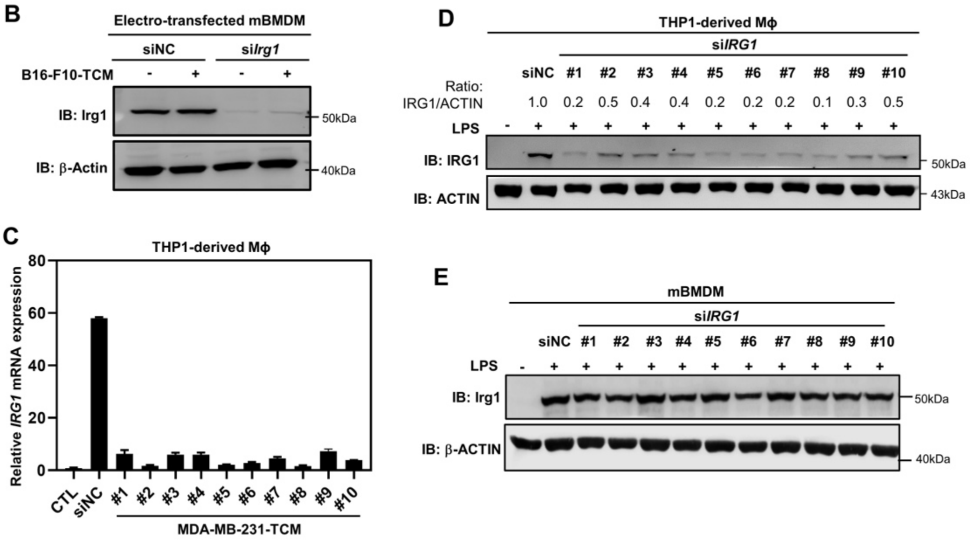

Fig. S1. Deletion of *IRG1* in macrophages.

(**A**) The siRNA sequences targeting mice and human *IRG1*, the targeted position and knockdown efficiency were summarized.

(**B**) Depletion of *Irg1* in mouse macrophages by siRNA. Briefly, si*Irg1* was transfected by electroporation in mBMDMs following the stimulation with B16-F10-TCM. Irg1 protein expression was determined by western-blot. Note: the electro-transfection led to macrophage activation, as evidenced by Irg1 induction in cells before the challenge with B16-F10-TCM.

(**C-D**) Depletion of *IRG1* in human macrophages by siRNA. Briefly, si*IRG1* was transfected by RNAiMAX in THP-1-derived macrophages following the challenge with either LPS or MDA-MB-231-TCM. The mRNA and protein expression of IRG1 was determined by qRT-PCR (C) and western-blot (D), respectively.

(**E**) siRNA targeting human *IRG1* was transected by RNAiMAX in mBMDMs following LPS challenge. Mouse Irg1 protein was determined by western-blot.

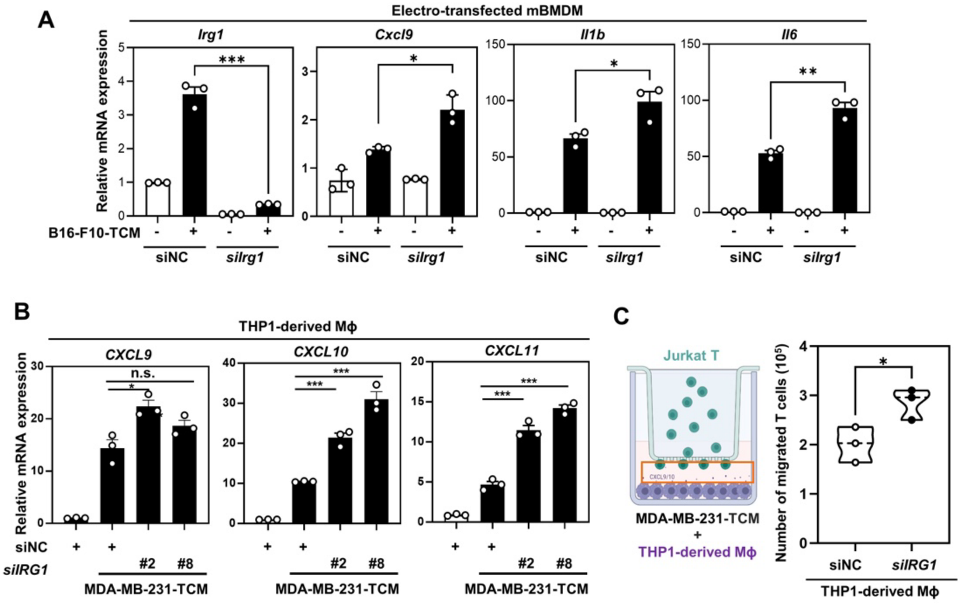

Fig. S2. Deletion of *IRG1* in macrophages promotes T cell trafficking.

(**A**) *Irg1* was depleted by siRNA mBMDMs, and the mRNA expression of indicated genes was examined by qRT-PCR in mouse macrophages after B16-F10-TCM stimulation for 12 hours.

(**B**) *IRG1* was depleted by siRNA in THP1-derived macrophages, and the mRNA expression of indicated genes was examined by qRT-PCR in human macrophages after MDA-MB-231-TCM stimulation for 12 hours.

(**C**) Macrophage and T cell co-culture transwell system. In brief, THP1-derived macrophages described in B were placed in the lower chamber. Meanwhile, Jurkat T cells were placed in the upper chamber and their migration into the lower chambers was counted.

Data are means ± SEM, and the p values were calculated by unpaired, two-tailed Student's t test. *$p < 0.05$, **$p < 0.01$, ***$p < 0.001$, and n.s., non-significant ($p \geqslant 0.05$).

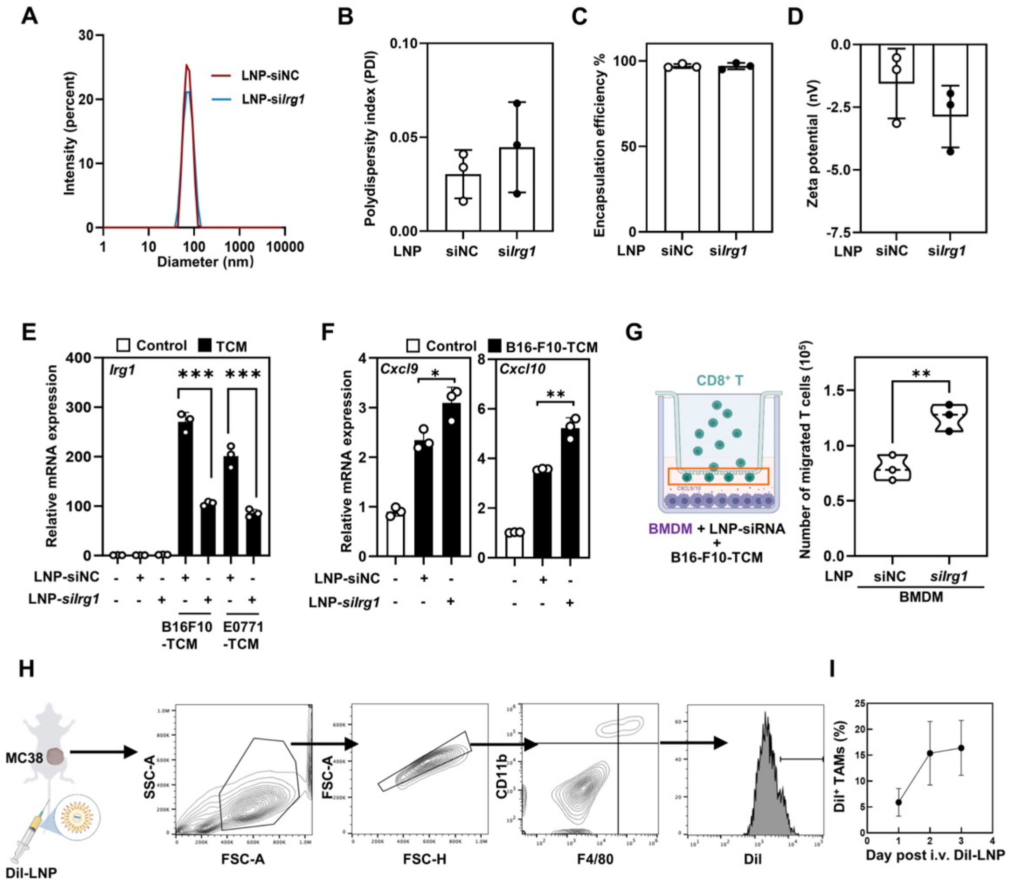

Fig. S3. LNP-based delivery of siRNA targeting *Irg1*.

(**A-D**) Biophysical parameters of indicated LNP-siRNA, including hydrodynamic diameter (A), polydispersity index (B), encapsulation efficiency (C) and Zeta potentials (D).

(**E-F**) LNP-si*Irg1* increased the expression of chemotaxis genes in activated macrophages. The knockdown efficiency of *Irg1* was confirmed by qRT-PCR in mBMDMs after stimulation with B16-F10-TCM or E0771-TCM for 12 hours (E). The mRNA expression of indicated chemotaxis genes was also determined in mBMDMs after stimulation with B16-F10-TCM for 12 hours (F).

(**G**) LNP-si*Irg1* facilitated $CD8^+$ T cells migration *in vitro*. Briefly, mBMDMs were treated with LNP-siRNA followed by stimulation with B16-F10-TCM for 12 hours in the lower chamber. Primary mouse $CD8^+$ T cells were placed in the upper chamber and their migration were counted.

(**H-I**) LNPs were labeled by DiI fluorescence and then intravenously injected into mice bearing MC38 colon cancer. The ratios of LNP-labeled TAMs were determined by flow cytometry.

Data are means ± SD for (A-F) and means± SEM for I. the p values were calculated by unpaired, two-tailed Student's t test. $*p < 0.05$, $**p < 0.01$, $***p < 0.001$, and n.s., non-significant ($p \geqslant 0.05$).

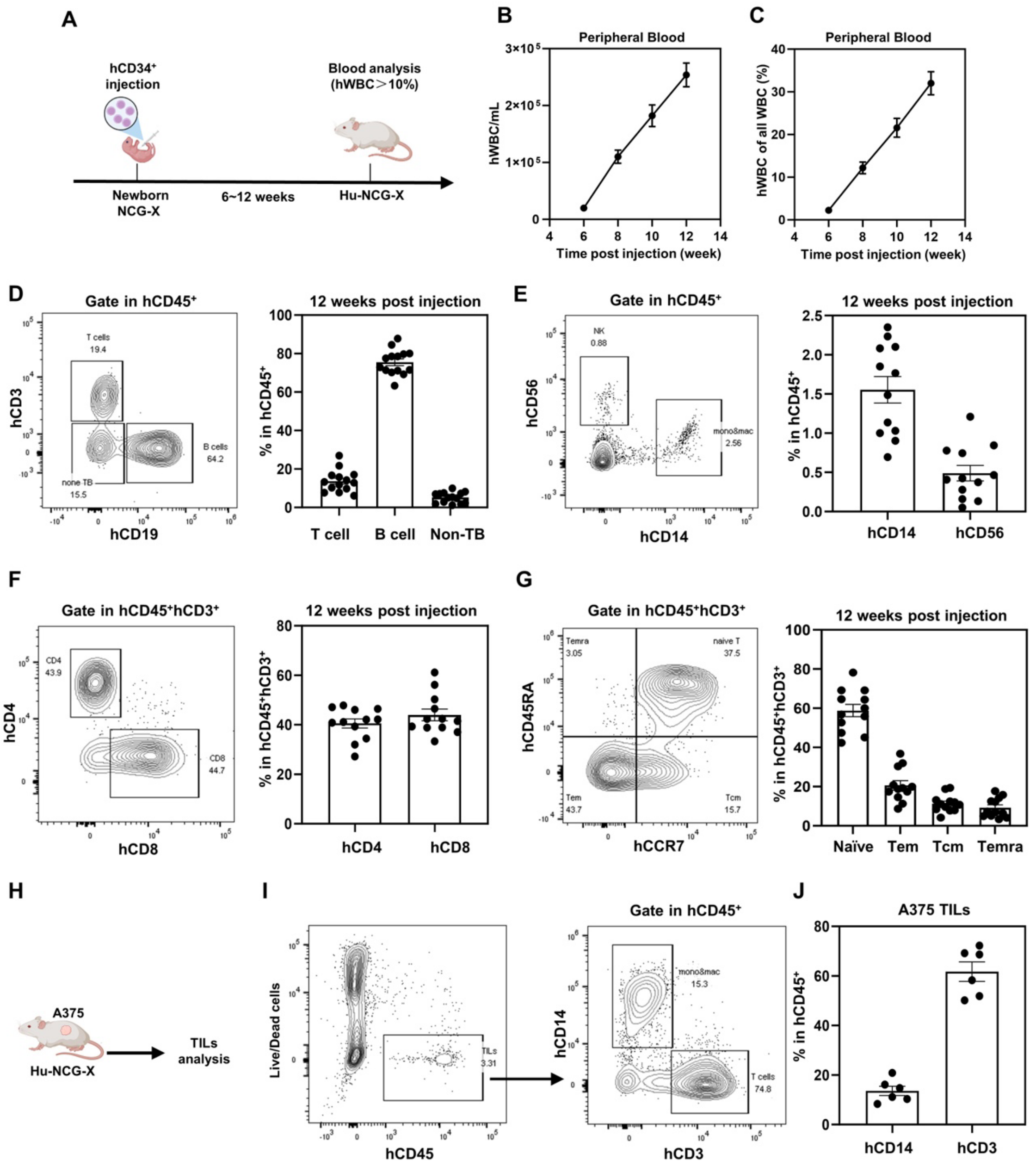

Fig. S4. Generation and characterization of HIS mice.

(**A**) Experimental schematics of the generation of HIS mice. Briefly, 3 to 7-day-old newborn NCG-X pups were intrahepatically injected with $CD34^{+}CD38^{-}$ cells isolated from human fetal liver. After 6-12 weeks, peripheral blood samples were collected followed by flow cytometry analysis to detect the reconstitution of human immune cells.

(**B-C**) The number (B) and proportion (C) of $CD45^{+}$ hWBCs in peripheral blood were detected and calculated by flow cytometry.

(**D-G**) The proportion of reconstituted human immune cells in peripheral blood (PB) of HIS mice were quantified by flow cytometry at 12 weeks after human $CD34^{+}CD38^{-}$ transplantation. The proportions of human T cells ($hCD3^{+}$), B cells ($hCD19^{+}$) and non-TB cells ($hCD3^{-}hCD19^{-}$)

in $hCD45^+$ cells were shown in D (n=14); The proportions of human mono & mac ($hCD14^+$) and NK cells ($hCD56^+$) in $hCD45^+$ cells were shown in E (n=12). The proportions of $hCD4^+$ and $hCD8^+$ T cells in $hCD3^+$ were shown in F (n=12); The proportions of indicated human T cell subsets in $hCD3^+$ was shown in G (n=12). Tem denotes effect memory T cells ($CCR7^-CD45RA^-$), Tcm denotes central memory T cells ($CCR7^+CD45RA^-$), Temra denotes effect memory T cells expressing CD45RA ($CCR7^-CD45RA^+$) and naïve denotes naïve T cells ($CCR7^+CD45RA^+$).

(**H**) Tumor-infiltrating human lymphocytes (TILs, $hCD45^+$) were analyzed in HIS mice bearing A375 melanoma.

(**I**) Representative plot showed the gating strategy of human mono & mac ($CD14^+$) and human T cells ($CD3^+$) in TILs.

(**J**) Quantification of human $CD14^+$ and $CD3^+$ cells in TILs of A375 melanoma (n=6). Data are means ± SEM.

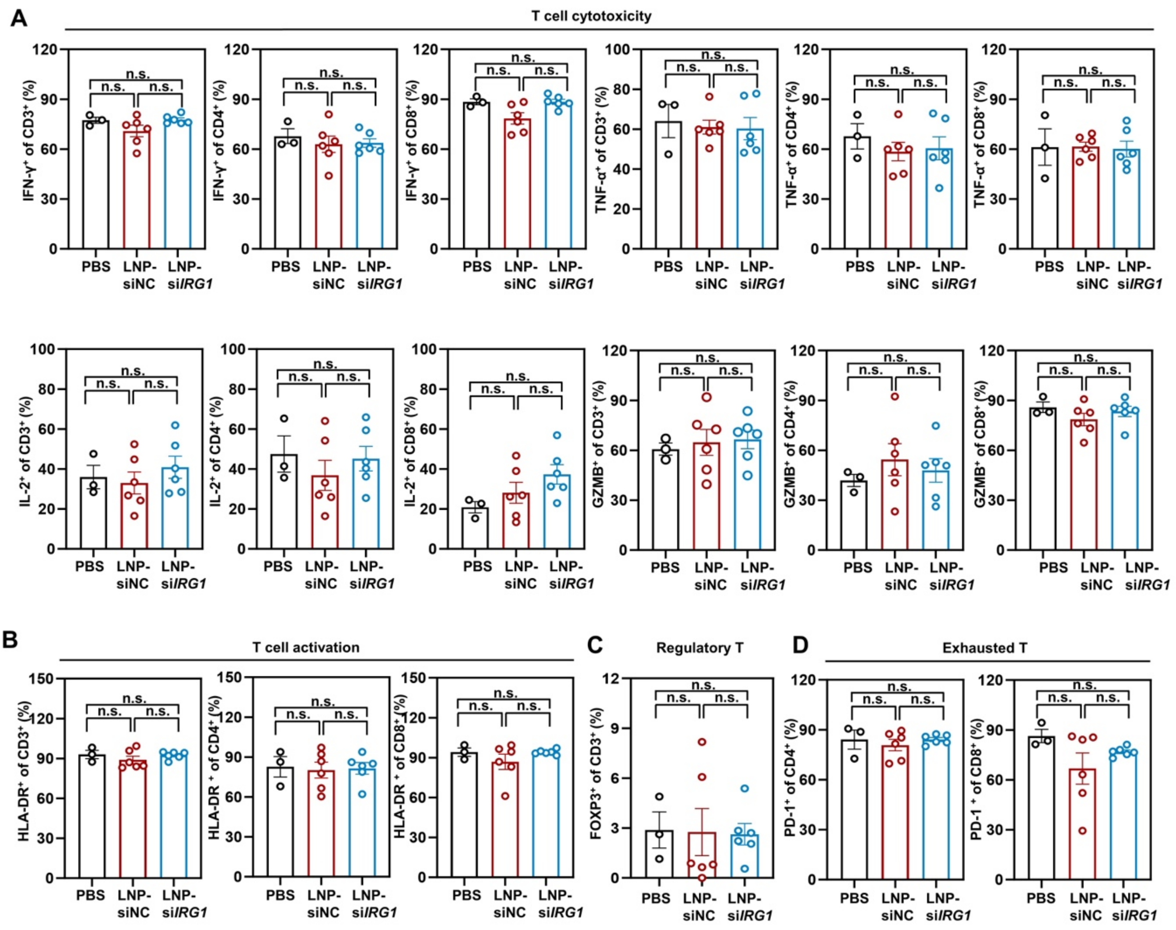

Fig. S5. LNP-based delivery of si*IRG1* does not change the functions of tumor-infiltrating T cells in HIS mice.

(**A**) The cytotoxicity of tumor-infiltrating T cells in HIS mice (n=3-6 per group) was quantified by the staining of IFN-γ, TNF-α, IL-2, and GZMB in $CD3^+$, $CD4^+$ and $CD8^+$ T cells, respectively. Related to Fig. 1D.

(**B**) The activation of tumor-infiltrating T cells in HIS mice was quantified by the staining of $HLA\text{-}DR^+$ in $CD3^+$, $CD4^+$ and $CD8^+$ T cells, respectively.

(**C**) The proportions of tumor-infiltrating regulatory T cells ($FOXP3^+$ in $CD3^+$) in HIS mice were quantified.

(**D**) $PD1^+$ T cells in HIS mice were quantified in $CD4^+$ and $CD8^+$, respectively. Data are means ± SEM, and the p values were calculated by one-way ANOVA. n.s. denotes non-significant ($p \geqslant 0.05$).

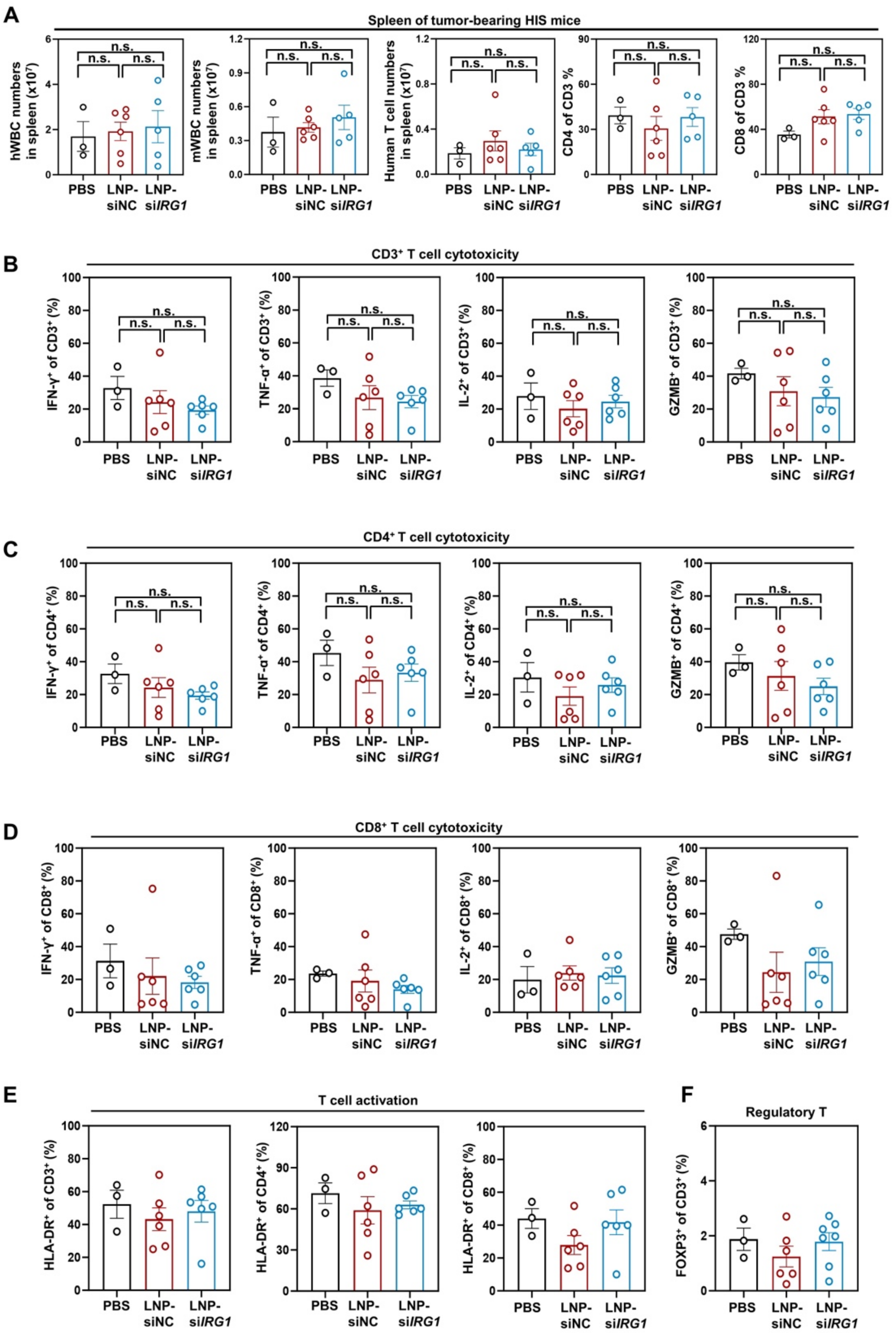

Fig. S6. LNP-based delivery of si*IRG1* does not alter human T cell function in the spleen of tumor-bearing HIS mice.

(**A**) Immune cells in the spleen of tumor-bearing HIS mice (n = 3-6 per group) were analyzed by flow cytometry. Numbers of $hCD45^+$, $mCD45^+$ and $hCD3^+$ and the proportions of $CD4^+$ or $CD8^+$ in $CD3^+$T cells were detected.

(**B-D**) The cytotoxicity of spleen T cells was quantified by the staining of IFN-γ, TNF-α, IL-2, and GZMB in $CD3^+$, $CD4^+$ and $CD8^+$ T cells, respectively.

(**E**) The activation of spleen T cells was quantified by the staining of HLA-DR$^+$ in CD3$^+$, CD4$^+$ and CD8$^+$ T cells, respectively.

(**F**) The proportion of regulatory T cells (FOXP3$^+$ in CD3$^+$) in spleen was quantified.

Data are means ± SEM, and the p values were calculated by one-way ANOVA. n.s. denotes non-significant ($p \geqslant 0.05$).

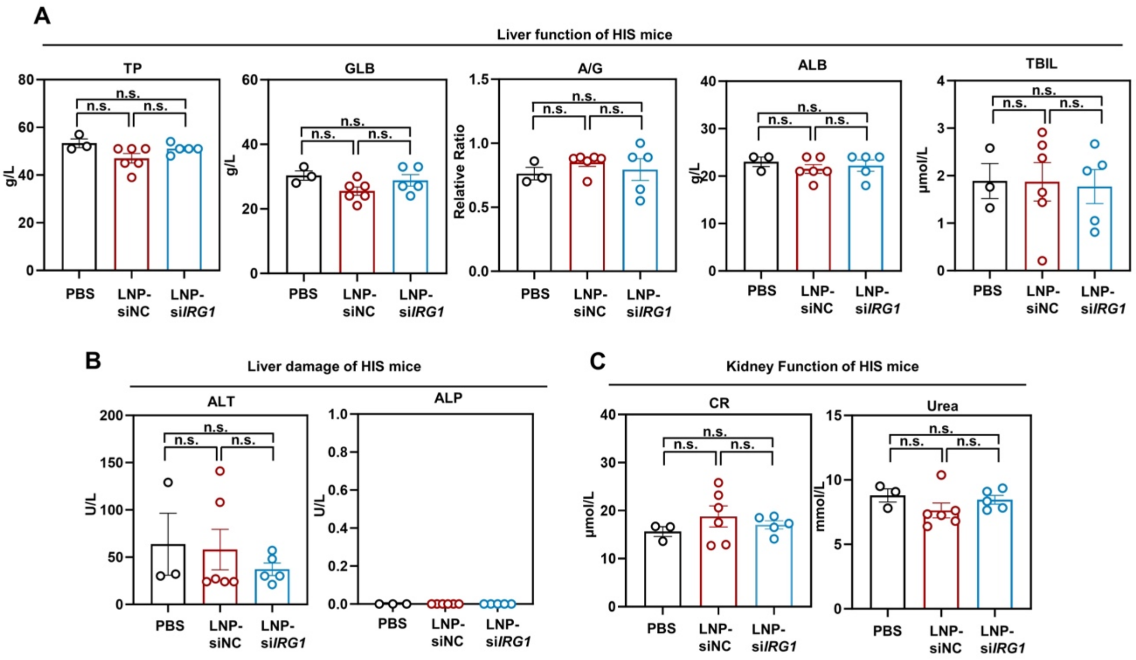

Fig. S7. LNP-based delivery of si*IRG1* does not cause tissue injury in HIS mice.

(**A-C**) Blood biochemistry analysis associated with liver function (A), liver damage (B) and kidney damage (C) were performed in HIS mice (n = 3-6 per group). Related to Fig. 2A.

TP: total protein, GLB: globin, A/G: ratio of albumin versus globin, ALB: albumin, TBIL: total bilirubin, ALT: alanine transaminase, ALP: alkaline phosphatase, CR: creatinine. Data are means ± SEM, and the p values were calculated by one-way ANOVA. n.s. denotes non-significant ($p \geqslant 0.05$).

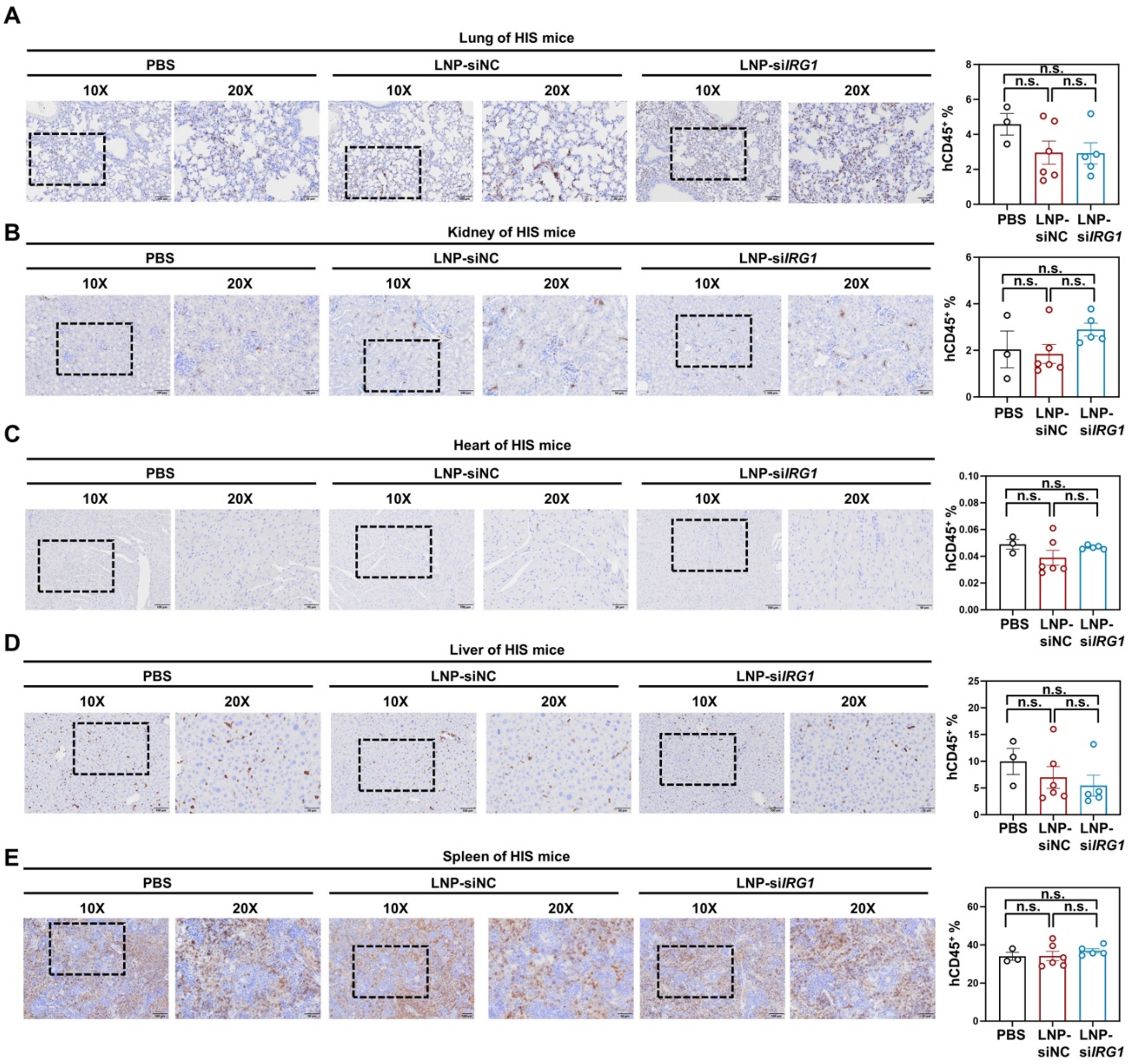

Fig. S8. LNP-based delivery of si*IRG1* does not induce human immune cell infiltration in tissues of HIS mice.

(**A-E**) Representative images of anti-hCD45 staining in different organs of HIS mice. Scale bars represent 100 μm (original magnification 10X) and 50 μm (original magnification 20X). The proportions of $hCD45^{+}$ in total cells of the indicated tissues were quantified. Data are means ± SEM, and the p values were calculated by one-way ANOVA. n.s., non-significant (p ⩾0.05).

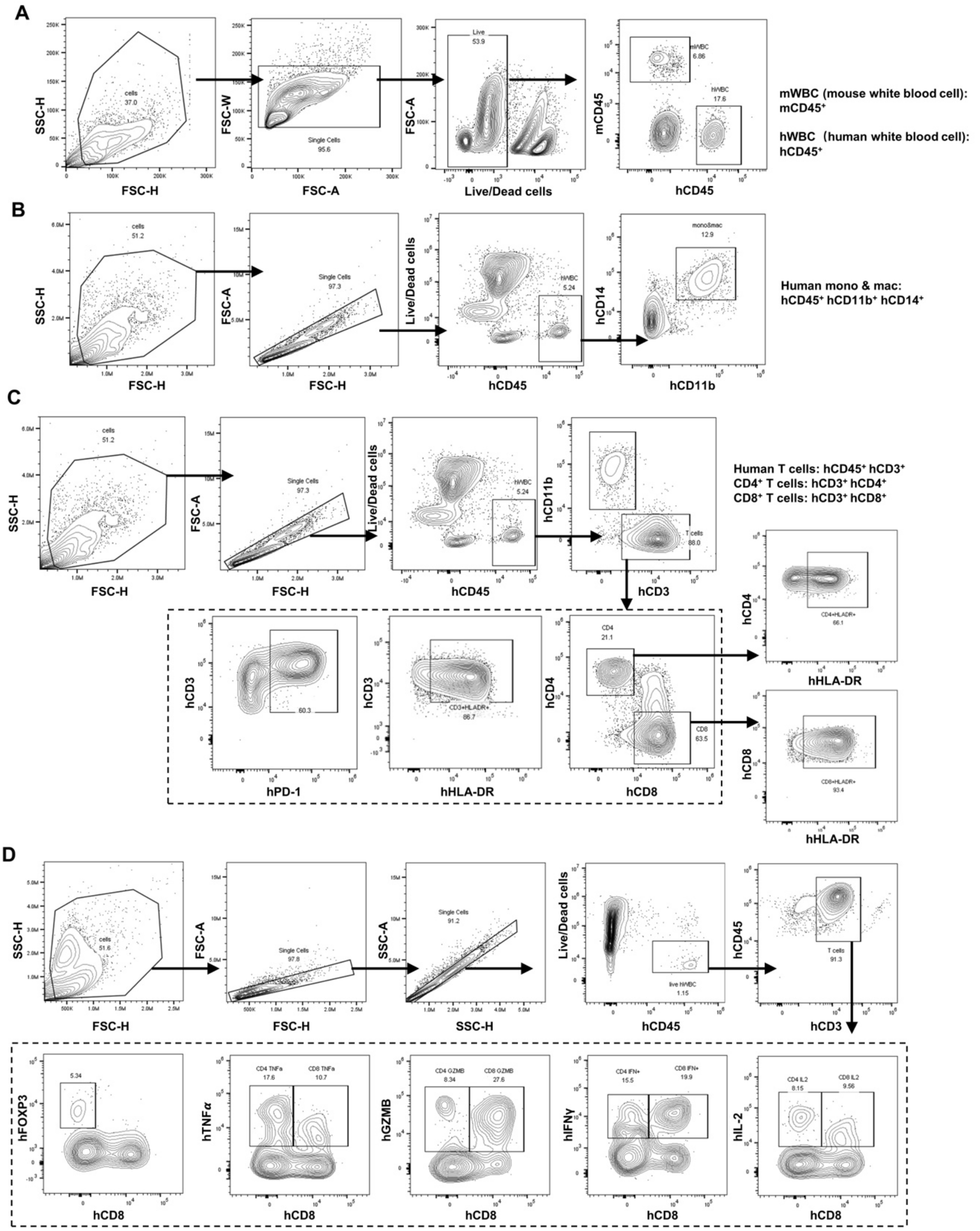

Fig. S9. Gating strategy for flow cytometry analysis.

(**A**) Gating strategy for human and mouse CD45^{+} WBCs.

(**B**) Flow cytometry-based isolation of human mono & mac cells for qRT-PCR analysis of *IRG1* mRNA expression. Related to Fig. 1L.

(**C**) Gating strategy for T cell activation and PD-1 expression in spleen and TILs.

(**D**) Gating strategy for T cell cytotoxicity and regulatory T cells in spleen and TILs.

Table S1. Sequences of primers used in this study

Gene	Sequence	Species
Irg1-F	TAAGAGCTTCCCGGCACATC	Mouse
Irg1-R	AATGACGGGCTTCATGCTCT	Mouse
Cxcl9-F	GAGCAGTGTGGAGTTCGAGG	Mouse
Cxcl9-R	TCCGGATCTAGGCAGGTTTG	Mouse
Cxcl10-F	AATGAGGGCCATAGGGAAGC	Mouse
Cxcl10-R	AGCCATCCACTGGGTAAAGG	Mouse
Il-1β-F	AGCTACGAATCTCCGACCAC	Mouse
Il-1β-R	CGTTATCCCATGTGTCGAAGAA	Mouse
Il-6-F	TAGTCCTTCCTACCCCAATTTCC	Mouse
Il-6-R	TTGGTCCTTAGCCACTCCTTC	Mouse
Actin-F	CACTGTCGAGTCGCGTCC	Mouse
Actin-R	TCATCCATGGCGAACTGGTG	Mouse
ACOD1-F	TGGGGCCTTTTATGCCAACT	Human
ACOD1-R	CTCACCTGTGGCCTGTTGAT	Human
ACTIN-F	CTTCGCGGGCGACGAT	Human
ACTIN-R	CCACATAGGAATCCTTCTGACC	Human
CXCL9-F	CCAGTAGTGAGAAAGGGTCGC	Human
CXCL9-R	AGGGCTTGGGGCAAATTGTT	Human
CXCL10-F	GTGGCATTCAAGGAGTACCTC	Human
CXCL10-R	TGATGGCCTTCGATTCTGGATT	Human
CXCL11-F	GACGCTGTCTTTGCATAGGC	Human
CXCL11-R	GGATTTAGGCATCGTTGTCCTTT	Human

Table S2. Antibodies used in this study

Application	Target	Species	Clone/Cat#	Supplier
Flow cytometry	CD45	Human	HI30	Biolegend
	CD3	Human	UCHT1	Biolegend
	CD14	Human	HCD14	Biolegend
	CD4	Human	RPA-T4	Biolegend
	CD8	Human	RPA-T8	BD bioscience
	CD56	Human	5.1H11	Biolegend
	CCR7	Human	3D12	eBioscience
	CD45RA	Human	HI100	BD bioscience
	HLA-DR	Human	L243	Biolegend
	PD-1	Human	EH12.2H7	Biolegend
	CD11b	Human&mouse	M1/70	Biolegend
	FOXP3	Human&mouse	150D/E4	eBioscience
	IL-2	Human	MQ1-17H12	Biolegend
	IFN-γ	Human	4S.B3	Biolegend
	TNF-α	Human	MAb11	Biolegend
	Granzyme B	Human&mouse	QA16A02	Biolegend
	CD45	mouse	30-F11	Biolegend
	F4/80	mouse	BM8	Biolegend
Western blot	IRG1	Human	D6H2Y	Cell Signaling Technology
	Irg1	Mouse	HA722819	Huabio

Reference

Akinc, A., Maier, M.A., Manoharan, M., Fitzgerald, K., Jayaraman, M., Barros, S., Ansell, S., Du, X., Hope, M.J., Madden, T.D., *et al.* (2019). The Onpattro story and the clinical translation of nanomedicines containing nucleic acid-based drugs. Nat Nanotechnol 14, 1084-1087.

Bajema, I.M., Wilhelmus, S., Alpers, C.E., Bruijn, J.A., Colvin, R.B., Cook, H.T., D'Agati, V.D., Ferrario, F., Haas, M., Jennette, J.C., *et al.* (2018). Revision of the International Society of Nephrology/Renal Pathology Society classification for lupus nephritis: clarification of definitions, and modified National Institutes of Health activity and chronicity indices. Kidney Int 93, 789-796.

Chen, Y.J., Li, G.N., Li, X.J., Wei, L.X., Fu, M.J., Cheng, Z.L., Yang, Z., Zhu, G.Q., Wang, X.D., Zhang, C., *et al.* (2023). Targeting IRG1 reverses the immunosuppressive function of tumor-associated macrophages and enhances cancer immunotherapy. Sci Adv 9, eadg0654.

Gauger, P.C., Vincent, A.L., Loving, C.L., Henningson, J.N., Lager, K.M., Janke, B.H., Kehrli, M.E., Jr., and Roth, J.A. (2012). Kinetics of lung lesion development and pro-inflammatory cytokine response in pigs with vaccine-associated enhanced respiratory disease induced by challenge with pandemic (2009) A/H1N1 influenza virus. Vet Pathol 49, 900-912.

Gibson-Corley, K.N., Olivier, A.K., and Meyerholz, D.K. (2013). Principles for valid histopathologic scoring in research. Vet Pathol 50, 1007-1015.

Kleiner, D.E., Brunt, E.M., Van Natta, M., Behling, C., Contos, M.J., Cummings, O.W., Ferrell, L.D., Liu, Y.C., Torbenson, M.S., Unalp-Arida, A., *et al.* (2005). Design and validation of a histological scoring system for nonalcoholic fatty liver disease. Hepatology 41, 1313-1321.

Li, H., Xiao, Y., Li, Q., Yao, J., Yuan, X., Zhang, Y., Yin, X., Saito, Y., Fan, H., Li, P., *et al.* (2022). The allergy mediator histamine confers resistance to immunotherapy in cancer patients via activation of the macrophage histamine receptor H1. Cancer Cell 40, 36-52 e39.

Li, Y., Masse-Ranson, G., Garcia, Z., Bruel, T., Kok, A., Strick-Marchand, H., Jouvion, G., Serafini, N., Lim, A.I., Dusseaux, M., *et al.* (2018). A human immune system mouse model with robust lymph node development. Nat Methods 15, 623-630.

Miao, E.A., Leaf, I.A., Treuting, P.M., Mao, D.P., Dors, M., Sarkar, A., Warren, S.E., Wewers, M.D., and Aderem, A. (2010). Caspase-1-induced pyroptosis is an innate immune effector mechanism against intracellular bacteria. Nat Immunol 11, 1136-1142.

Ren, D., Liu, W., Ding, S., and Li, Y. (2022). Protocol for generating human immune system mice and hydrodynamic injection to analyze human hematopoiesis in vivo. STAR Protoc 3, 101217.

Standage, S.W., Waworuntu, R.L., Delaney, M.A., Maskal, S.M., Bennion, B.G., Duffield, J.S., Parks, W.C., Liles, W.C., and McGuire, J.K. (2016). Nonhematopoietic Peroxisome Proliferator-Activated Receptor-alpha Protects Against Cardiac Injury and Enhances Survival in Experimental Polymicrobial Sepsis. Crit Care Med 44, e594-603.
